# Encoding of Physics Concepts: Concreteness and Presentation Modality Reflected by Human Brain Dynamics

**DOI:** 10.1371/journal.pone.0041784

**Published:** 2012-07-27

**Authors:** Kevin Lai, Hsiao-Ching She, Sheng-Chang Chen, Wen-Chi Chou, Li-Yu Huang, Tzyy-Ping Jung, Klaus Gramann

**Affiliations:** 1 Institute of Education, National Chiao-Tung University, Hsinchu City, Taiwan, Republic of China; 2 Institute for Neural Computation, University of California San Diego, San Diego, California, United States of America; 3 Institute of Psychology and Ergonomics, Technical University Berlin, Berlin, Germany; Tel Aviv University, Israel

## Abstract

Previous research into working memory has focused on activations in different brain areas accompanying either different presentation modalities (verbal vs. non-verbal) or concreteness (abstract vs. concrete) of non-science concepts. Less research has been conducted investigating how scientific concepts are learned and further processed in working memory. To bridge this gap, the present study investigated human brain dynamics associated with encoding of physics concepts, taking both presentation modality and concreteness into account. Results of this study revealed greater theta and low-beta synchronization in the anterior cingulate cortex (ACC) during encoding of concrete pictures as compared to the encoding of both high and low imageable words. In visual brain areas, greater theta activity accompanying stimulus onsets was observed for words as compared to pictures while stronger alpha suppression was observed in responses to pictures as compared to words. In general, the EEG oscillation patterns for encoding words of different levels of abstractness were comparable but differed significantly from encoding of pictures. These results provide insights into the effects of modality of presentation on human encoding of scientific concepts and thus might help in developing new ways to better teach scientific concepts in class.

## Introduction

In the past decade, researchers have become more interested in the implications of neuroscience research on education and have tried to incorporate brain imaging results to the educational field [1]. Investigating how various materials are learned, previous neuroimaging studies have compared activation patterns in distinct brain areas either elicited by pictures as compared to words [2], or concrete as compared to abstract words [3]. However, previous working-memory studies focused on non-scientific concepts and none of them has investigated how humans encode different scientific concepts. Among the different scientific concepts, physics is known to be one of the most difficult subject domains for students in school [4]. Physics is difficult for students to learn because the majority of concepts in physics are abstract, making it difficult to form an image (for instance, inertia, gravity, etc.). However, visualizing concepts that are taught primarily in an oral fashion facilitates encoding and further processing of the same concept as an additional modality contributes to a deeper processing of the content [5].

Baddeley's [6] well-known model of working memory highlights the distinction between verbal and visual processing and is composed of the phonological loop (responsible for maintaining verbal information through articulatory rehearsal) and the visuo-spatial sketchpad (for holding and manipulating visuospatial representations) as two slave systems besides a central-executive. Researchers have used neuroimaging techniques, such as functional magnetic resonance imaging (fMRI), to study such distinctions in the presentation modality and the associated cognitive processes and have reported verbal working memory processes to be more lateralized to the left hemisphere [2], whereas spatial content is predominantly processed in the right hemisphere [7]. This is in line with studies reporting stronger activation in left frontal cortex during encoding of words [8], and stronger activation in the right frontal cortex during encoding of pictures [9]. However, other studies reported increased bilateral activations during a verbal working memory task [10]–[11]. Although these results in general suggest that the left hemisphere provides the neural basis for the phonological loop while the right hemisphere underlies the visuo-spatial sketchpad [12], the findings are controversial.

Researchers have also used high temporal-resolution EEG recordings to study the distinction of verbal and visual working memory. Unlike other neuroimaging methods with high spatial resolution (e.g. fMRI), EEG allows for investigating the neural basis of cognitive processes in the range of milliseconds. An EEG study by Hwang and colleagues reported symmetrically distributed bilateral activity for both kinds of working memory tasks, verbal and visual [13]. Their results showed that verbal stimuli elicited more theta-band oscillation, as compared to nonverbal stimuli. More specifically, verbal tasks elicited significantly greater power bilaterally in the theta band, over frontal and occipital areas in the alpha and beta bands [13]. Onton et al. [14] reported increased frontal midline theta and low-beta activity during a visual working memory task. Raghavachari [15] used a verbal working memory task and reported increased theta activity at the beginning of a trial. The author also reported widely distributed brain regions generating theta activity, consistent with other EEG studies that showed increased theta synchronization between frontal and posterior regions during a working memory task [16].

In addition to the distinction between words and pictures, researchers have further categorized words as either *abstract* or *concrete*. According to Paivio's [5] dual coding theory (DCT), concrete words have an advantage over abstract words (known as the concreteness effect) because they can be processed using two systems (verbal and visual). Researchers have found the left frontal area to be involved in categorizing nouns as either concrete or abstract [17], [18]. Binder et al's [19] fMRI study investigated brain activation patterns involved in processing of nonwords, concrete and abstract words. Compared to nonwords, both concrete and abstract words activated the left side of the brain. However, Kounios and Holcomb's [3] argued that brain activation for concrete words is more pronounced in the right hemisphere, which is assumed to be related to the image system of the brain.

The present study investigates modulations in different frequency bands accompanying encoding of physics concepts of varying concreteness and modalities in a modified Sternberg task. Analyzing the power spectrum of the EEG has shown that power in the theta band (4–8 Hz) increases with greater mental effort or cognitive challenge [20], [21]. Tasks that are associated with modulation of theta power include successful vs. unsuccessful memory encoding [22] and working memory load [20]. Many studies indicated that the most pronounced theta activity emanates from near dorsal anterior cingulate cortex (ACC) [23], and that theta activity is most pronounced during encoding [24]. Raghavachari et al. [15] noted that theta oscillation is not limited to the frontal area but is distributed throughout the brain, including posterior areas. It also has been reported that alpha (8–12 Hz) power is often seen to decrease under conditions of greater attentional demand, primarily at posterior sites [25]. In occipital areas, increased alpha activity indicates a resting, idling state, and alpha suppression occurs when a person is processing visual information [26]. Studies have shown that alpha suppression occurs when a task becomes more demanding and requires greater cognitive effort [20]. However, alpha oscillations in the parietal areas have generated mixed findings. Some studies have shown decreases in parietal alpha [25], while other studies have found an increase in alpha oscillations [27].

In general, there is a lack of studies that focus on scientific concepts and how these are processed in working memory. To close this gap, we investigated the brain dynamics accompanying encoding, rehearsal, and retrieval of physics concepts. Particularly, we examined whether EEG oscillatory patterns accompanying encoding and retrieval of pictorial physics concepts would be comparable to those associated with encoding and retrieval of concrete and abstract verbal physics concepts. To this end, we compared the human brain dynamics in three conditions (pictures, high imageable words, and low imageable words) (1) to determine the brain dynamics and any asymmetry accompanying processing of pictorial versus verbal physics concepts and (2) to investigate whether the power in different frequency bands differ between concrete and abstract physics concepts.

## Methods

### Subjects

Sixty-three undergraduate students participated in this study. All participants were right-handed and had normal or corrected-to-normal vision. All participants were volunteer students who were paid for their participation. The Institutional Review Board of the China Medical University Hospital approved the study. All participants were asked to read and sign a consent form regarding the process of the experiment. All the students have taken physics courses at high school and passed college entrance examination in physics; thus they were familiar with the physics concepts.

### Experiment and Procedure

**Figure 1:**
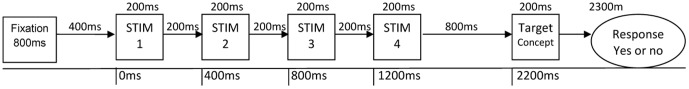
Picture, high imageable word, and low imageable word. The words were originally in Chinese during the study.

To examine how students encode physics-related concepts, this study used a modified Sternberg [28] paradigm. Participants were asked to remember a number of sequentially presented physics concepts for a short retention period. The concepts were presented in three different conditions, either as pictures, high imageable words, or low imageable words, with 60 different physical concepts for each condition. The pictures were physics concepts with concrete attributes, which were supposed to be processed in form of images of the concepts by the participants (e.g., turning direction of gearwheels). The high imageable word condition included physics concepts with concrete attributes, presented as words. Stimuli in this word category were selected to be easily encoded and remembered as pictures (e.g., the words “pendulum”, “gear wheel”). The low imageable word condition consisted of physics concepts with abstract attributes that were difficult to recode into images (e.g., the words “inertia,” “static equilibrium,” “average power”). Figure 1 shows samples of high and low imageable words. This task allowed us to study working memory processes of encoding, rehearsing, and retrieving physics concepts, particularly, how different attributes of physics concepts (concrete and abstract) in two different presentation forms (word and picture) are encoded.

**Figure 2:**
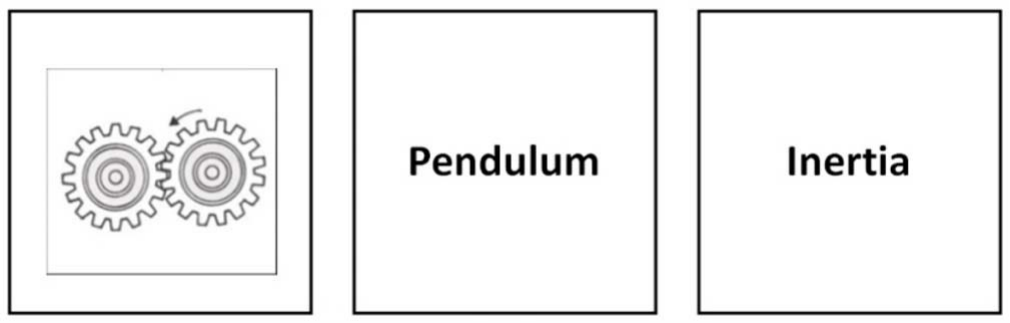
Encoding Task timeline.

Each trial included four stimuli of one condition (picture, high imageable words, low imageable words) and one target probe, with stimulus presentation duration of 200 ms for each presentation. The target was presented 800 ms after the last stimulus was presented, and participants responded to whether the target had appeared in the previous 4 stimuli (Figure 2). Subjects were asked to respond “Yes” by pressing the left mouse button, and “No” by pressing the right mouse button. Each condition consisted of 60 trials with conditions separated by a 30- second rest period. For each trial, 4 of the 60 concepts were chosen as stimuli and presented sequentially, and each of the 60 scientific concepts appeared as the target concept once. The target was present in the memory set 70% of the time. Response time was defined as the time duration between the target's onset and the participants' response.

### Data Recording

The EEG was recorded continuously using a Neuroscan Sim-Amp 2 system (Neuroscan, El Paso, Texas) with 66 electrodes mounted in an elastic cap. Electrodes were positioned according to the extended 10–20 system and referenced to linked mastoids. Vertical (vEOG) and horizontal (hEOG) eye movements were recorded using four separate bipolar electrodes. EEG signals were collected at a sampling rate of 1,000 Hz with an analog band pass from 0.01 to 100 Hz. Inter-electrode impedance was kept below 5 k Ohms.

### Data Analysis

For the EEG data, intervals containing extreme peak-to-peak deflections or large bursts of high frequency electromyographic activity were excluded from further analysis by visual inspection. Eye movement activity was not removed. Data were analyzed by custom MATLAB scripts built on the open source EEGLAB toolbox [29] (http://sccn.ucsd.edu/eeglab). After digitally filtering to remove frequencies below 0.5 Hz and above 50 Hz and downsampling to 250 Hz, the data were submitted to extended infomax Independent Component Analysis (ICA) [30] using the *runica* function [31] from the EEGLAB toolbox. Default extended-mode runica training parameters were used with the stopping weight change set to 1e–7 or 1 e–8.

#### Component Selection

Independent component (IC) activation time series for each subject were categorized as brain activity or nonbrain activity by visual inspection of their activation spectra, time courses, and scalp topographies. Subsequently, an equivalent current dipole model was computed for each selected brain IC using a boundary element head model (BEM) as implemented in the DIPFIT toolbox [32]. ICs with bilaterally distributed scalp maps were fit with a dual equivalent dipole model with a positional symmetry constraint. Only ICs with equivalent dipole models accounting for more than 85% of actual IC scalp map variance and ICs with an equivalent dipole model located inside the head sphere were included in further analysis.

#### Component Power Spectra and Event-related Spectral Perturbation

After ICA decomposition, the data were separated into non-overlapping epochs of 5.7 sec, which included the stimulus and probe onsets. Each epoch was taken 1 sec before the onset of the first stimulus to 4.7 sec after the onset, giving sufficient time to include subjects' response. IC activation for each trial was then transformed into a spectrographic image using three-cycle Morlet wavelets in a frequency range between 3 and 50 Hz. Spectrographic images were composed of mean event-related spectral perturbation (ERSP) images by converting to log power, subtracting mean log power from a 1000-msec baseline interval drawn from before the presentation of the first stimulus, and then averaging trials for each condition [33].

#### Independent Component Clustering

The selected ICs from all the subjects were then clustered using a K-means clustering algorithm as implemented in EEGLAB. Each of the measures (spectrum, ERP, ERSP, inter-trial coherence, and scalp topography) save dipole location (with only 3 dimensions) was compressed into a 10-dimensional vector. Subsequently, all measures were combined and further compressed by principal component analysis (PCA) into a single 25-dimensional cluster position vector for each IC. Dipole location was given a weight of 20, ERSP was given a weight of 9, and other measures (spectrum, ERP, inter-trial coherence, and scalp topography) were given a weight of 1. IC's whose distance to the cluster centroid was greater than three standard deviations away from the cluster centroid were removed. ICs were clustered into 40 clusters. Subsequently, visual inspection of single ICs contributing to the respective cluster mean led to the exclusion of ICs with power spectra indicative of muscle activity and ICs whose dipole locations were questionable with respect to the cluster mean dipole location.

#### ERSP Statistics

Significant changes in power from the mean spectral baseline for each component over the time course of a trial were computed using bootstrap resampling [29]. To visualize power modulations in the frequency range from 3 to 50 Hz over time, we subtracted the mean baseline log power spectrum from each spectral estimate, producing the baseline-normalized ERSP. Nonsignificant time–frequency points were masked with zero values in the mean ERSPs and displayed as green. Significant differences with respect to baseline activity were displayed in red and blue for positive and negative deviations from the baseline activity, respectively. Condition differences were computed using EEGLAB study with bootstrap statistical thresholds of p<0.005 for frontal midline cluster and p<0.001 for parietal and occipital clusters.

## Results

### Behavioral Data

Participants' responses were correct in more than 80% of all trials, indicating that they were able to perform the task and that they paid attention to the stimuli during the experiment. Averaged reaction times were 680.80 (SD = 108.33), 669.43 (SD = 97.27) and 667.75 (SD = 98.50) ms for pictures, high imageable words, and low imageable words, respectively. A repeated measures ANOVA for the reaction times in the three conditions revealed no significant effect, *F*_2, 124_ = 1.62, p>0.05.

### Mean spectral power changes

#### Frontal Midline Cluster

**Figure 3:**
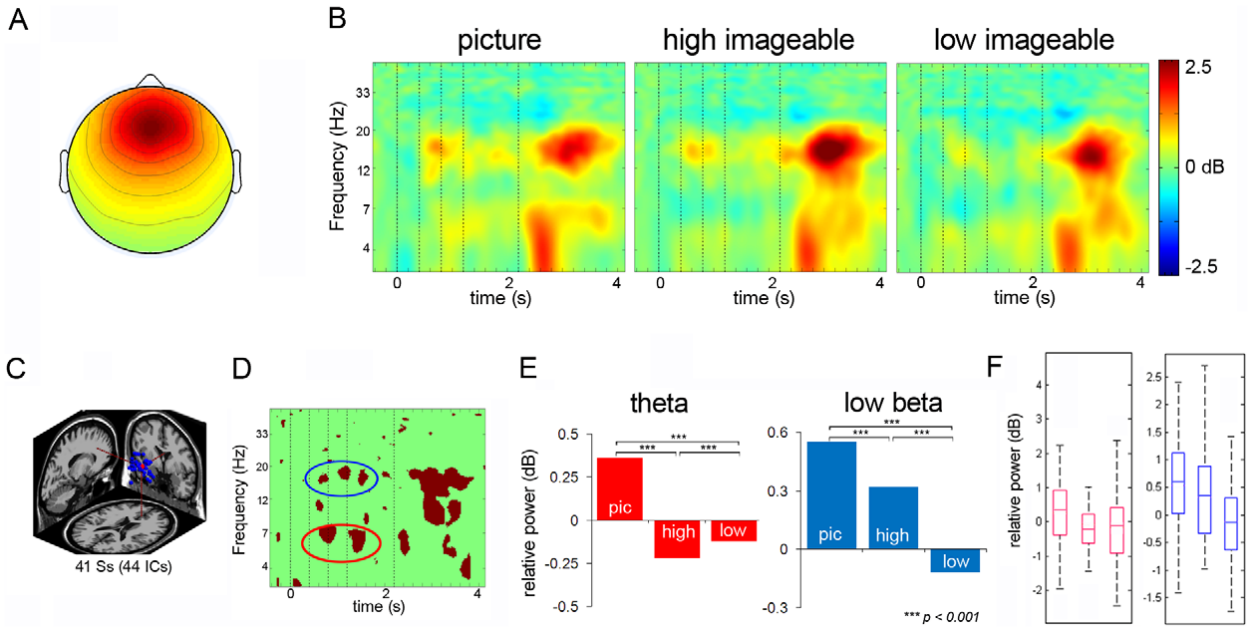
Frontal Midline Cluster. Panel A: Scalp map for frontal midline cluster. Panel B: ERSP's from frontal midline cluster (dotted lines signify onset of stimuli). Panel C: dipole location for frontal midline cluster. Panel D: Significant differences across the three conditions. Panel E: Post-hoc comparison between the conditions in theta bands and low beta bands. Panel F: Box plots showing the range and probability distribution of the power values.

Fig. 3b shows average ERSPs for an IC cluster with a centroid located in or near the ACC over the time course of all trials including presentation of the stimuli, the probe, and the final response. The frontal midline cluster comprised 44 IC's from 41 subjects. The average scalp maps for these clusters along with the dipole locations are presented in Figure 3a and 3c. The ERSP revealed theta and beta power increases following the onsets of stimuli. When the probe appeared, the frontal midline cluster exhibited a burst of theta power, followed by an increase in beta power.

All non-green pixels of the ERSP difference image (Fig. 3d) across three conditions (picture, high imageable words, low imageable words) were statistically significant (p<0.005). To quantitatively analyze spectral differences among the three conditions, we calculated the mean spectral power values in the time windows indicating significant differences between the conditions as defined by the bootstrapped difference ERSPs (depicted in red for the masked ERSP's) for each of the three conditions. Subsequently, an ANOVA was computed to compare theta power revealing statistically significant differences among the three conditions (F_2, 1560_ = 1229.92, p<0.001). Tukey HSD test indicated that the mean theta power for the picture condition was significantly greater compared to high imageable words (p<0.001) and low imageable words (p<0.001) (Fig. 3e). In addition, theta power with encoding of low imageable words was more pronounced than that with encoding of high imageable words (p<0.001) (Table 1). For the low-beta frequency range, the results indicated a significant difference among the three conditions (pictures, high imageable words, and low imageable words) (F_2, 909_ = 435.66, p<0.001) (Table 1). Tukey HSD tests revealed low-beta power for the picture condition to be significantly stronger than that for the high imageable words (p<0.001) and low imageable words (p<0.001), as well as that for high imageable words as compared to low imageable words (p<0.001) (Table 1).

**Table 1:** Descriptive statistics and ANOVA results for theta and low beta band in frontal midline cluster.

Name	N	Mean	SD	F	p	Post-hoc
Theta				*F* (2, 1560)		
Picture	521	.36	.11	1229.92***	<0.001	Picture>Low Imageable***
High Imageable	521	−.22	.10			Picture>High Imageable***
Low Imageable	521	−.12	.32			Low Imageable>High Imageable***
Low Beta				*F* (2, 909)		
Picture	304	.55	.31	435.66***	<0.001	Picture>High Imageable***
High Imageable	304	.32	.24			Picture>Low Imageable***
Low Imageable	304	−.12	.29			High Imageable>Low Imageable***

*** p<0.001, **p<0.01, *p<0.05.

#### Left and Right Parietal clusters

**Figure 4:**
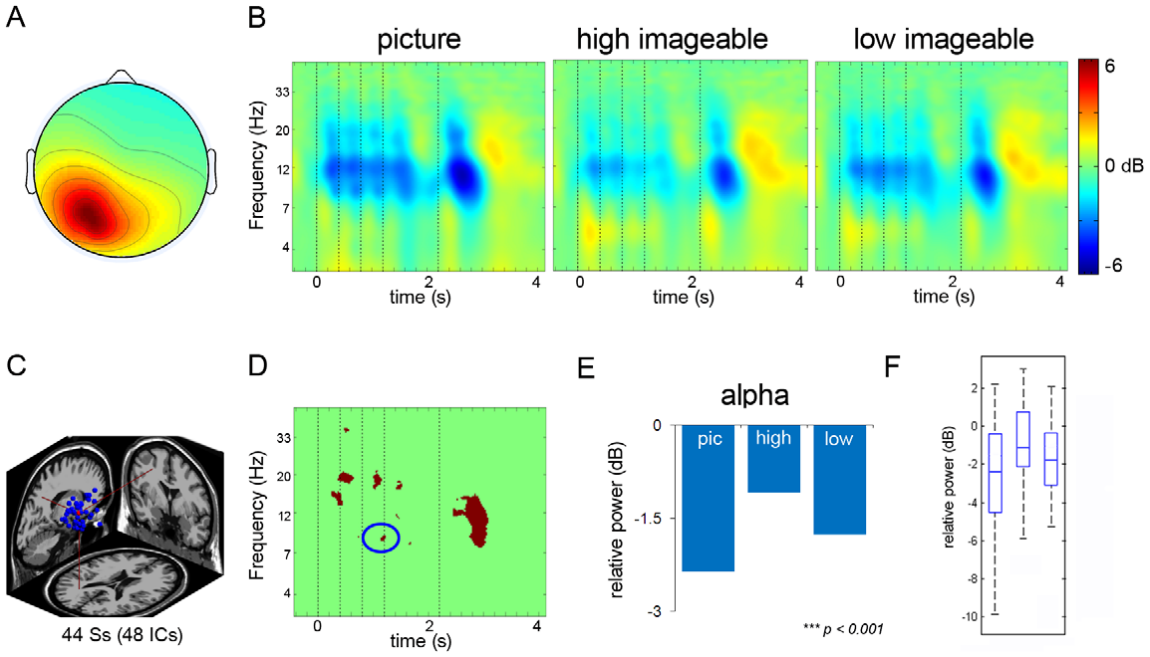
Left Parietal Cluster. Panel A: Scalp map for left parietal cluster. Panel B: ERSP's from left parietal cluster (dotted lines signify onset of stimuli). Panel C: Dipole location for left parietal cluster. Panel D: Significant differences across the three conditions. Panel E: Post-hoc comparison between the conditions in left parietal alpha band. Panel F: Box plot showing the range and probability distribution of the power values.

**Figure 5:**
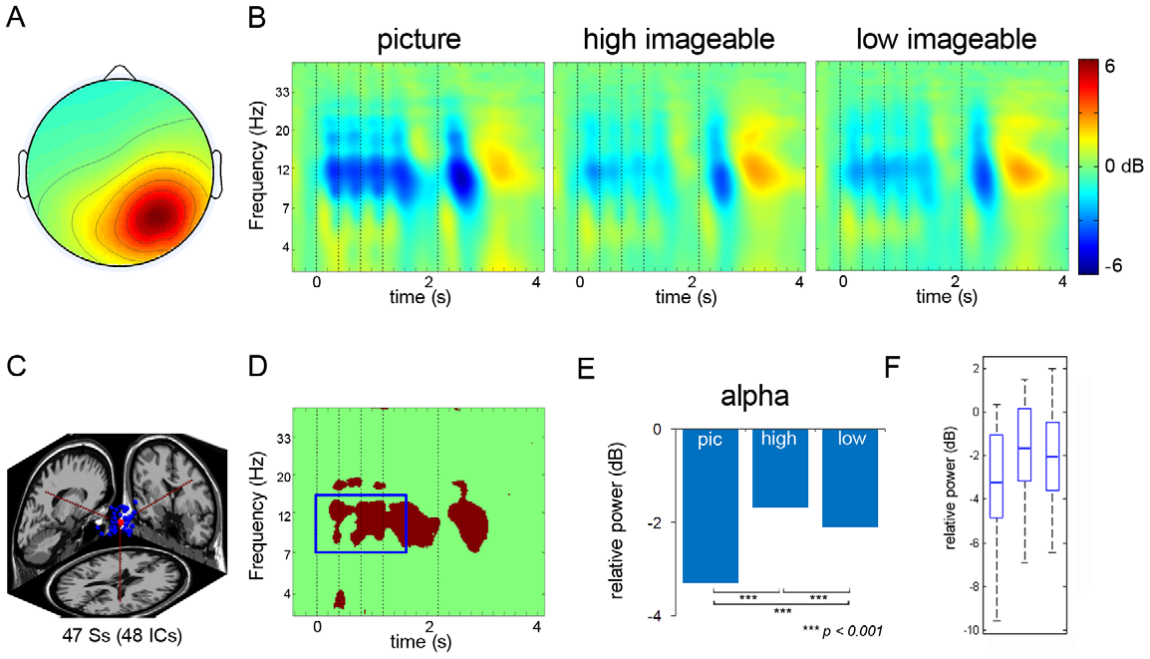
Right Parietal Cluster. Panel A: Scalp map for right parietal cluster. Panel B: ERSP's from right parietal cluster (dotted lines signify onset of stimuli). Panel C: Dipole location for right parietal cluster. Panel D: Significant differences across the three conditions. Panel E: Post-hoc comparison between the conditions in right parietal alpha band. Panel F: Box plot showing the range and probability distribution of the power values.

A cluster with the cluster centroid located in or near the left parietal cortex included 48 IC's from 44 subjects. A right parietal cluster included 48 IC's from 47 subjects. Fig. 4b and Fig. 5b show average ERSP images of the left and right parietal clusters, respectively. The ERSP revealed theta power increase after onset of the first stimulus, followed by alpha power decrease with each consecutive stimulus. The stimulus induced theta-power increase was apparent for the first stimulus but not the subsequent stimuli. For the left parietal cluster, there was a greater theta-power increase after onset of the first stimulus for both high and low imageable words, as compared to the picture conditions. In contrast, there was a slightly greater theta power increase after the first stimulus in picture as compared to both high and low imageable words conditions in the right parietal cluster. Further, there was greater alpha suppression after stimulus onset of pictures compared to the onset of high and low imageable words, both for the right and left parietal clusters. With onset of the probe concept, high imageable words in the left parietal cluster elicited greater theta power compared to the other two conditions; for the right parietal cluster; in contrast, the pictures elicited greater theta power compared to the two word conditions. After the response, the left and right parietal components cluster both revealed a strong burst in alpha-band power.

Statistical comparisons of power values within the time periods as depicted by bootstrapping for the difference ERSP revealed significant differences in alpha power across the conditions for the left parietal cluster (F_2, 42_ = 753.54, p<0.001) (Table 2). Tukey HSD tests showed that alpha power for the picture condition was significantly lower than that for the high imageable (p<0.001) and low imageable word conditions (p<0.001). In addition, the low imageable word condition was accompanied by significantly less alpha power compared to the high imageable words condition (p<0.001) (Table 2). For the right parietal cluster, the ANOVA revealed significant differences across the conditions for alpha power (F_2, 5256_ = 4337.39, p<0.001) (Table 3). Tukey HSD tests showed that the mean alpha power for the picture condition was significantly lower than that of high imageable word condition (p<0.001) and low imageable word condition (p<0.001), and alpha power in the low imageable word condition was significantly lower than high imageable (p<0.001) (Fig. 5e).

**Table 2:** Descriptive statistics and ANOVA results for alpha band in left parietal cluster.

Name	N	Mean	SD	F(2, 42)	p	Post-hoc
Picture	15	−2.36	.11	753.54***	<0.001	|Picture|>|High Imageable|***
High Imageable	15	−1.09	.08			|Picture|>|Low Imageable|***
Low Imageable	15	−1.82	.08			|Low Imageable|>|High Imageable|***

*** p<0.001, **p<0.01, *p<0.05.

**Table 3:** Descriptive statistics and ANOVA results for alpha band in right parietal cluster.

Name	N	Mean	SD	F(2, 5256)	p	Post-hoc
Picture	1753	−3.29	.43	4337.39***	<0.001	|Picture|>|Low Imageable|***
High Imageable	1753	−1.69	.37			|Picture|>|High Imageable|***
Low Imageable	1753	−2.10	.27			|Low Imageable|>|High Imageable|***

*** p<0.001, **p<0.01, *p<0.05.

#### Left and Right Occipital Cluster

**Figure 6:**
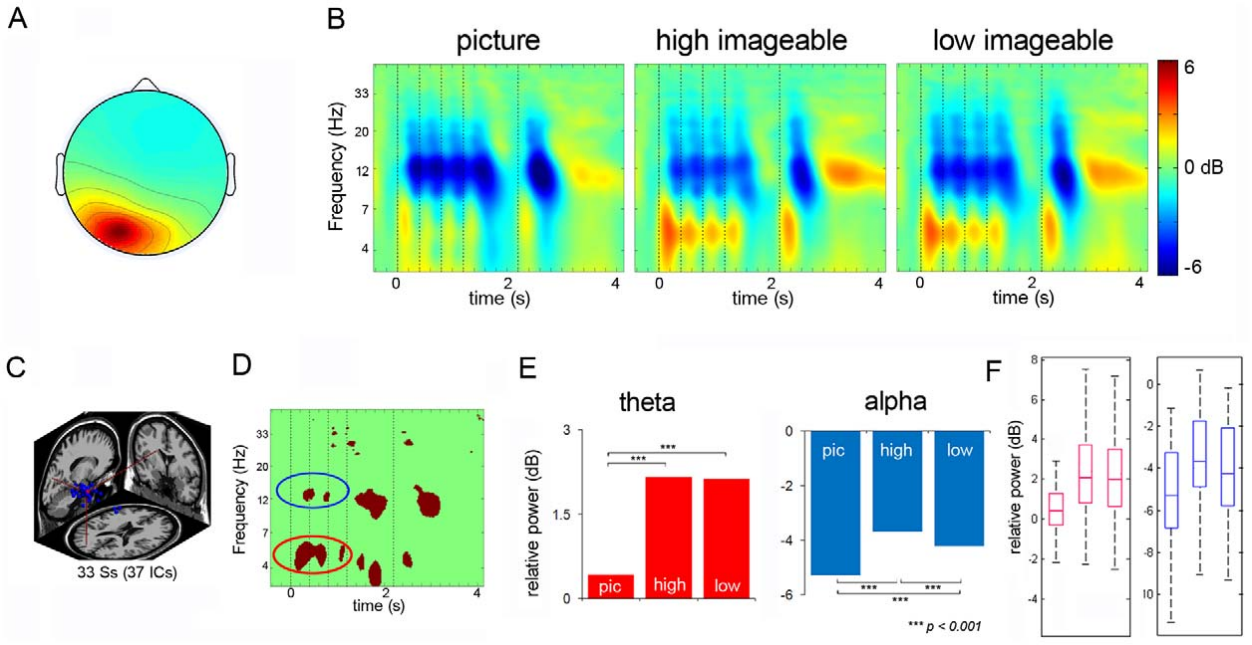
Left Occipital Cluster. Panel A: Scalp map for left occipital cluster. Panel B: ERSP's from left occipital cluster (dotted lines signify onset of stimuli). Panel C: Dipole location for left occipital cluster. Panel D: Significant differences across the three conditions. Panel E: Post-hoc comparison between the conditions in theta band and alpha band. Panel F: Box plots showing the range and probability distribution of the power values.

**Figure 7:**
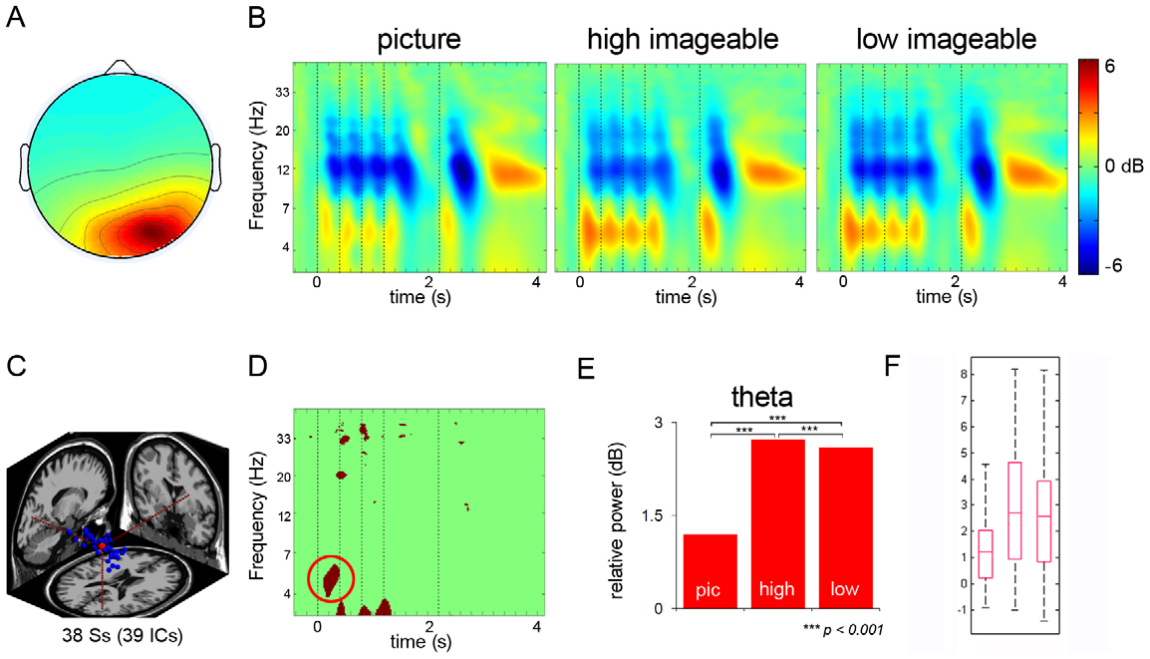
Right Occipital Cluster. Panel A: Scalp map for right occipital cluster. Panel B: ERSP's from right occipital cluster (dotted lines signify onset of stimuli). Panel C: Dipole location for right occipital cluster. Panel D: Significant differences across the three conditions. Panel E: Post-hoc comparison between the conditions in theta band. Panel F: Box plot showing the range and probability distribution of the power values.

The left occipital cluster included 37 IC's from 33 subjects, and the right occipital cluster included 39 IC's from 38 subjects. Fig. 6b and Fig. 7b show average ERSP images of the left and right occipital clusters, respectively. The analyses revealed a precise theta-power augmentation, followed by alpha-power suppression across the three conditions that corresponded to the onsets of the presentation of each stimulus at 0, 400, 800, and 1200 ms. Power in the theta band was most pronounced for the first stimulus, and then decreased with each subsequent stimulus. For both left and right occipital clusters, there was a greater theta-power increase on both high and low imageable words compared to the picture condition (Fig. 6e). In contrast, there was greater alpha suppression following pictures than that following the high and low imageable words, both in the right and left occipital clusters (cf. Fig. 6e). The results also showed that the picture condition exerted greater theta power than the word conditions in the right occipital cortex, as compared to the left occipital. When the probe concept appeared, the high and low imageable words were associated with increased power in the theta band compared to the picture condition in both left and right occipital clusters. Also, the picture condition in the left cluster showed greater alpha suppression than the other two conditions. After button press, the left and right occipital components clusters showed a strong alpha desynchronization.

For the left occipital cluster, the ANOVA comparing theta power revealed significant differences across the conditions (F_2, 2154_ = 1160.08, p<0.001) (Table 4). Tukey HSD tests indicated that the mean theta power for the high and low imageable words was significantly greater than that in the picture condition (all ps<0.001) (Table 4). In the alpha band, significant differences were found between the three conditions (F_2, 459_ = 664.40, p<0.001) (Table 4). Tukey HSD tests revealed the mean alpha-power suppression for the picture condition to be more pronounced than that of the high (p<0.001) and low imageable word conditions (p<0.001), and the suppression in the low imageable words was greater than that in the high imageable words (p<0.001) (Table 4). For the right occipital cluster, the ANOVA found significant differences in the theta power across the three conditions (F_2, 765_ = 1213.89, p<0.001) (Table 5). Tukey HSD tests indicated that the mean theta power for the high and low imageable words condition was significantly higher than that of the picture condition (all ps<0.001), and high imageable word condition was associated with significantly higher theta power than the low imageable word condition (p<0.001) (Table 5).

**Table 4:** Descriptive statistics and ANOVA results for theta and alpha bands in left occipital cluster.

Name	N	Mean	SD	F	p	Post-hoc
Theta				*F*(2, 2154)		
Picture	719	.42	.66	1160.08***	<0.001	Picture<High Imageable***
High Imageable	719	2.16	.85			Picture<Low Imageable***
Low Imageable	719	2.12	.82			
Alpha				*F*(2, 459)		
Picture	154	−5.29	.40	664.40***	<0.001	|Picture|>|Low Imageable|***
High Imageable	154	−3.68	.37			|Picture|>|High Imageable|***
Low Imageable	154	−4.22	.42			|Low Imageable|>|High Imageable|***

*** p<0.001, **p<0.01, *p<0.05.

**Table 5:** Descriptive statistics and ANOVA results for theta band in right occipital cluster.

Name	N	Mean	SD	F(2, 765)	p	Post-hoc
Picture	256	1.19	.40	1213.89	<0.001	Picture<High Imageable***
High Imageable	256	2.73	.40			Picture<Low Imageable***
Low Imageable	256	2.60	.39			High Imageable>Low Imageable***

*** p<0.001, **p<0.01, *p<0.05.

## Discussion

This study investigated EEG dynamics during a working memory task involving physics concepts presented in three different modalities: pictures, high imageable words and low imageable words.

### Stimulus-induced EEG dynamics

During the stimulus presentation, the anterior cingulate cortex exhibited theta and low-beta power augmentation in all three conditions, consistent with the results reported in Onton et al [14] in a verbal working memory task and Hwang et al [13] in both their verbal and nonverbal working memory tasks. The increase in both the theta and beta band could be interpreted as reflecting the need to sustain attention while encoding verbal and pictorial material into the specific working memory sub-components.

The left and right parietal cortex exerted theta-power augmentation after the first stimulus onset, followed by a pronounced alpha-power suppression with each subsequent stimulus presentation. Independent components with equivalent dipoles in or near the occipital cortices also exhibited a theta-power augmentation after onset of the first stimulus, followed by alpha-power suppression for each subsequent stimulus. Raghavachari et al. [15] reported that theta activity during working memory tasks was widely distributed across different brain regions, which was corroborated by Sarnthein et al [16], reporting increased theta synchronization between posterior and frontal regions. These results are in line with our findings of theta-power augmentation in the frontal, occipital and parietal cortices.

Though these previous studies supported the involvement of theta, alpha and beta modulations in the verbal and non-verbal working memory tasks, none of the studies have shown such detailed brain dynamics in the parietal and occipital areas as we have reported. The ERSP showed precise temporal dynamics of theta augmentation, followed by alpha suppression time-locked to the onset of the presentation of each stimulus. Theta synchronization was also seen to increase with task demands and working memory processes [15]. Alpha power is often seen to be decreased in tasks requiring greater demand and cognitive effort, primarily at posterior sites [20]. One possible reason for the precise pattern of theta increase and alpha decrease might be attributed to the physics-concepts related working memory task being more demanding and requiring more attention of participants.

### Effects of presentation modalities on the EEG

This study found that both high and low imageable words exerted greater theta power in both right and left occipital areas as compared to pictures, and this was more pronounced in the left than in the right hemisphere. A similar pattern was found in the parietal lobe. Our results replicate previous findings suggesting that theta oscillations play a role in more demanding tasks and especially in verbal working memory [15]. Studies have also proposed that theta activity in the left posterior areas was involved in verbal processing [34], [35]. Our results showed that the strength of theta burst decreased over successive stimulus presentations and that this modulation was more pronounced in the left as compared to right hemisphere. This could be attributed to the first stimuli requiring the most effort in encoding, as Raghavachari et al. [15] suggested that a possible role of theta oscillation in working memory was to rapidly encode information directly into long-term memory by synaptic modification or to synchronize different regions of the cortex that participated in the task [16].

In the occipital areas, it is well known that increased alpha activity indicates a resting state, and that there is alpha-power suppression when a person is viewing visual stimuli [26]. Our results indicated greater alpha suppression during encoding of pictures as compared to encoding of high and low imageable words in the posterior regions. We speculated that pictures might need more visual processing than words, and thus greater alpha suppression was found for pictures [15]. Our results also showed that high imageable words exerted slightly more theta augmentation than low imageable words in both the right and left occipital components, which is consistent with previous studies [17]. However, our study showed that EEG activities elicited by high and low imageable words in the working memory task in general were quite comparable, but significantly different from those elicited by the pictures.

Although the implications of our neuroimaging results to education are still preliminary, the results of this study might have some useful pedagogical implications. In general, we can assume from previous investigations that posterior theta is implicated in episodic memory and the encoding of items, and frontal midline theta is related to working memory and sustained effort. Our results suggest that verbal material requires greater involvement of episodic memory, while pictorial material requires more attention during encoding. This might be used to improve learning of scientific concepts in the classroom settings in terms of the sequencing of presentations of the concepts. Experimental results showed that words might pose a greater difficulty for students to learn initially, suggesting that presenting pictures and graphics first might be a good way for students to learn scientific concepts or presenting pictures/graphics along with words to lower the memory load and facilitate students in learning concepts. This is also supported by other previous studies [36].

### Conclusion

This study explored EEG dynamics in a working memory task involving physics concepts and compared spectral differences during stimulus presentation of different materials: pictures, high imageable words, and low imageable words. Results showed that the processing of high and low imageable words was quite comparable during encoding; however, we observed differences between the picture and the word conditions in several frequency bands and brain areas. The midline frontal clusters showed greater theta and low-beta activation during the presentation of the picture conditions. The activations following pictures, high and low imageable words were consistent with previous research that showed bilateral theta augmentation and alpha suppression in both hemispheres for verbal encoding, including both high and low imageable words. Though high imageable words and pictures all shared the same attributes of concrete physics concepts, their EEG oscillatory patterns differed largely. This sheds new light on how humans encode information and that encoding is greatly affected by the modality of presentation, but not so much by the conceptual attribute itself. The ERSP results provide new insights into students' encoding of scientific concepts, and more studies involving these kinds of concepts should be warranted in further research.
